# Supplementary material for: Correlation Analysis between Muskrat (Ondatra zibethicus) Musk and Traditional Musk
Source: Animals (Basel). 2023 May 18;13(10):1678. doi: 10.3390/ani13101678 (PMC10215723; doi:10.3390/ani13101678)
Supplement: Supplementary file 1 [file animals-13-01678-s001.zip › Table S2.pdf]

Supplementary Table S2. Metabolites of top20 in WM and BM

No.	WM			BM		
	**Chemical name**	**CAS**	**Molecular formula**	**Chemical name**	**CAS**	**Molecular formula**
1	Palmitic acid	57-10-3	$C_{16}H_{32}O_2$	Carbobenzyloxy -L-leucine degr	2018-66-8	$C_{14}H_{19}NO_4$
2	1-Hexadecanol	36653-82-4	$C_{16}H_{34}O$	Succinic acid	110-15-6	$C_4H_6O_4$
3	Succinic acid	110-15-6	$C_4H_6O_4$	Hippuric acid	495-69-2	$C_9H_9NO_3$
4	Behenic acid	112-85-6	$C_{22}H_{44}O_2$	Threo-beta-hyrdoxyaspartate	7298-99-9	$C_4H_7NO_5$
5	Glycine	56-40-6	$C_2H_5NO_2$	Palmitic acid	57-10-3	$C_{16}H_{32}O_2$
6	Stearic acid	57-11-4	$C_{18}H_{36}O_2$	Behenic acid	112-85-6	$C_{22}H_{44}O_2$
7	Oleic acid	112-80-1	$C_{18}H_{34}O_2$	4-Oxoproline	2002-02-0	$C_5H_7NO_3$
8	5-Aminovaleric acid	660-88-8	$C_5H_{11}NO_2$	Hypoxanthine	68-94-0	$C_5H_4N_4O$
9	Thymidine	50-89-5	$C_{10}H_{14}N_2O_5$	Methyl trans-cinnamate	103-26-4	$C_{10}H_{10}O_2$
10	Putrescine	110-60-1	$C_4H_{12}N_2$	Glycine	56-40-6	$C_2H_5NO_2$
11	4-Oxoproline	2002-02-0	$C_5H_7NO_3$	Biuret	108-19-0	$C_2H_5N_3O_2$
12	Lignoceric acid	557-59-5	$C_{24}H_{48}O_2$	D-Arabitol	488-82-4	$C_5H_{12}O_5$
13	2-Hydroxypyridine	142-08-5	C_5H_5NO	Stearic acid	57-11-4	$C_{18}H_{36}O_2$
14	Octadecanol	112-92-5	$C_{18}H_{38}O$	4-Hydroxyphenyl acetic acid	156-38-7	$C_8H_8O_3$
15	D-Arabitol	488-82-4	$C_5H_{12}O_5$	Oleic acid	112-80-1	$C_{18}H_{34}O_2$
16	Alanine	56-41-7	$C_3H_7NO_2$	Arachidic acid	506-30-9	$C_{20}H_{40}O_2$
17	Glucoheptonic acid	23351-51-1	$C_7H_{14}O_8$	Glycerol	56-81-5	$C_3H_8O_3$
18	4-Hydroxyphenylacetic acid	156-38-7	$C_8H_8O_3$	1-Octanal	124-13-0	$C_8H_{16}O$
19	2,4-Diaminobutyric acid	305-62-4	$C_4H_{10}N_2O_2$	Lignoceric acid	557-59-5	$C_{24}H_{48}O_2$
20	Serine	56-45-1	$C_3H_7NO_3$	Alanine	56-41-7	$C_3H_7NO_2$
