# Supplementary figures and images for: Correlation Analysis between Muskrat (Ondatra zibethicus) Musk and Traditional Musk
Source: Animals (Basel). 2023 May 18;13(10):1678. doi: 10.3390/ani13101678 (PMC10215723; doi:10.3390/ani13101678)

a

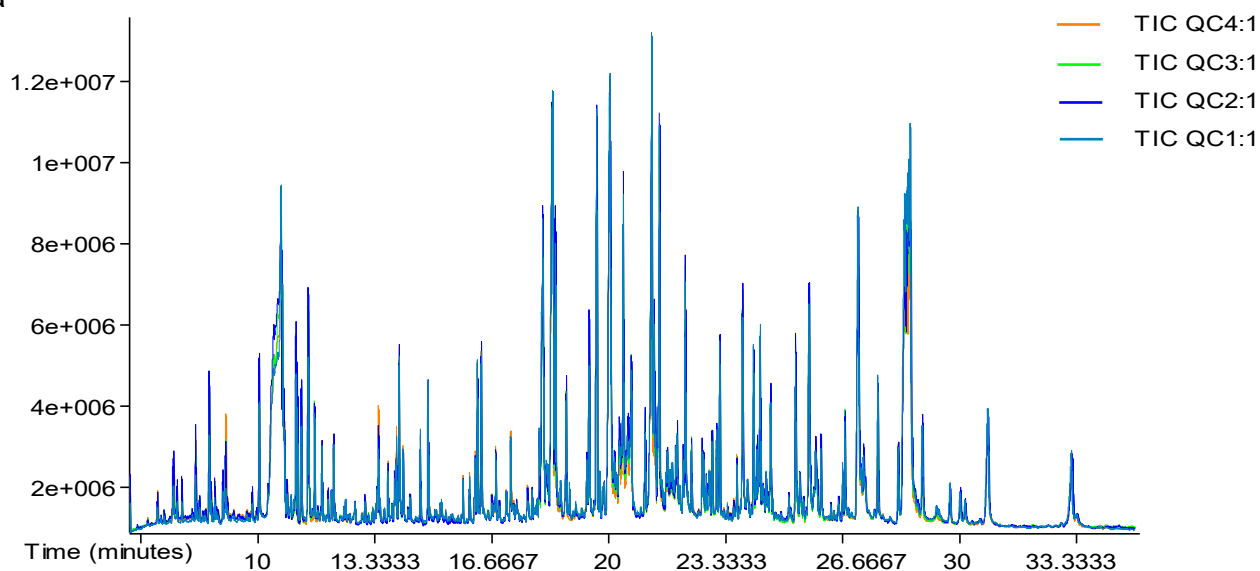

b

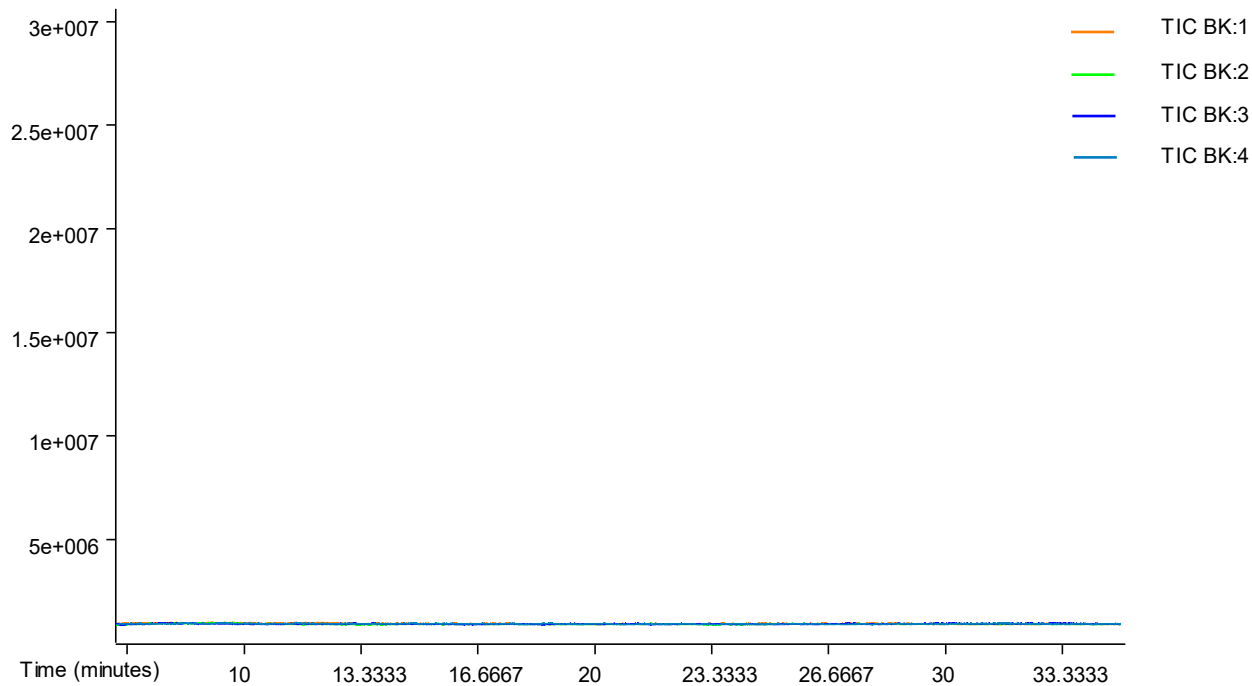

c

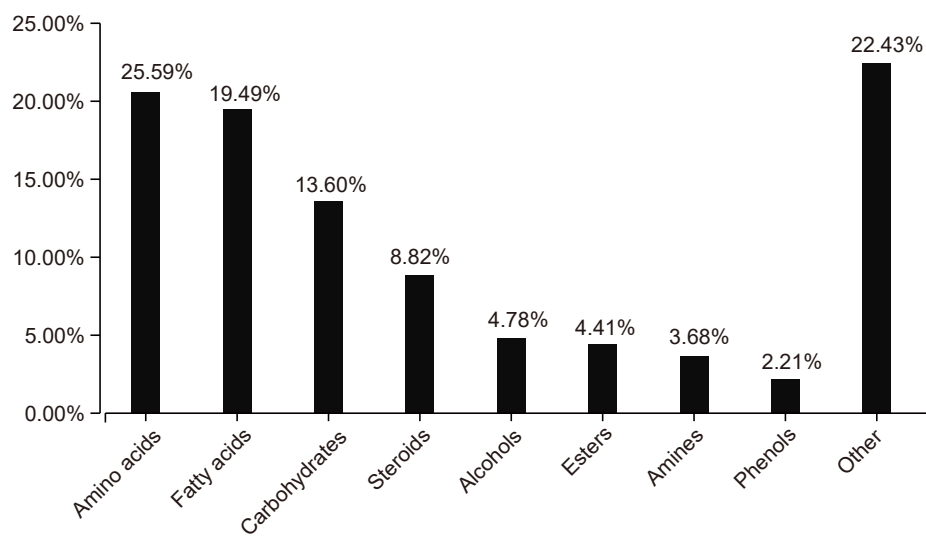

Supplement: Supplementary file 1 [file animals-13-01678-s001.zip › Figure S1.pdf]
